# Editorial: Epigenetic and metabolic regulators of breast carcinogenesis

**DOI:** 10.3389/fonc.2024.1383043

**Published:** 2024-03-28

**Authors:** Iman Mamdouh Talaat, Maha Saber-Ayad, Hauke Busch, Noha Mousaad Elemam

**Affiliations:** ^1^ Department of Clinical Sciences, College of Medicine, University of Sharjah, Sharjah, United Arab Emirates; ^2^ Research Institute for Medical and Health Sciences, University of Sharjah, Sharjah, United Arab Emirates; ^3^ Division for Medical Systems Biology, Lübeck Institute of Experimental Dermatology, Universität zu Lübeck, Lübeck, Germany; ^4^ University Cancer Center Schleswig-Holstein, University Hospital Schleswig-Holstein, Lübeck, Germany

**Keywords:** breast cancer, epigenetics, cancer metabolism, carcinogenesis, DNA methylation, miRNA

## 1 Introduction

This editorial features the articles published in this Research Topic in Frontiers in Oncology, which aimed to uncover the different epigenetic aspects and metabolic processes involved in breast tumorigenesis. The article by Jia et al. assessed the predictive capacity of fluorine-18 fluorodeoxyglucose positron emission tomography/computed tomography (18 F-FDG PET/CT) in prognostic risk assessment of invasive breast cancer (BC) patients. In a retrospective analysis of 91 patients undergoing preoperative 18 F-FDG PET/CT, radiomic signatures (RSs) were identified, and a radiomic score (Rad-score) was computed. The Rad-score, along with other factors, was independently associated with progression-free survival and overall survival. The clinicopathologic-radiomic-based model outperformed single clinical or radiomic models, exhibiting good predictive performance and enhanced individualized prognosis estimation. Therefore, integrating clinicopathological risks with Rad-score provides a robust method for prognostic evaluation in invasive BC patients, enhancing the accuracy of outcome predictions.

In the study by Pan et al., the authors explored the origin, molecular and pathological characteristics, treatment, and prognosis of claudin-low BC (CLBC). They highlighted that CLBC displays a higher histological grade and a greater likelihood of spreading to lymph nodes compared to other subtypes. Moreover, it is often associated with increased invasiveness as well as a less favorable prognosis and a lower likelihood of complete remission for CLBC. Hence, this aims to contribute to a comprehensive understanding and lay the groundwork for personalized BC treatments. This could contribute to a comprehensive understanding and lay the groundwork for personalized BC treatments.

From an epigenetic perspective, a study explored another aspect associated with BC progression and epithelial-mesenchymal transition (EMT) by identifying EMT-associated target genes (ETGs) of miR-222-3p. Their bioinformatic analysis showed that miR-222-3p might be a specific biomarker of basal-like BC. Furthermore, 10 core ETGs of miR-222-3p were identified where some of these genes might be useful diagnostic and prognostic biomarkers. The comprehensive analysis of these 10 ETGs and miR-222-3p indicated that they might be involved in the development of BC, shedding light on their potential as therapeutic targets for BC treatment (Fang et al.). On the other hand, the study by Zhang et al. developed a prognostic model for BC based on RNA guanine-7 methyltransferase (RNMT), FAM103A1, and 12 related microRNAs. Utilizing data from The Cancer Genome Atlas and TargetScan, a risk prognosis model accurately predicts 1-, 3-, 5-, and 10-year survival rates (>0.7 AUC). Such a model was linked to immune infiltration, suggesting potential immunotherapeutic targets for BC.

Down Syndrome (DS) patients present a unique cancer profile with a low risk of solid tumors but a higher risk of leukemia. A study by Bejaoui et al. explored DNA methylation and epigenetic aging in DS individuals with and without BC. Using the Infinium Methylation EPIC BeadChip array, differentially methylated sites in DS individuals with BC (T21-BC) were identified and linked to gene expression changes. Enriched processes included serine-type peptidase activity, epithelial cell development, GTPase activity, bicellular tight junction, and Ras protein signal transduction. Interestingly, epigenetic age acceleration analysis revealed no difference between T21-BC and DS individuals without BC (T21-BCF). This pioneering research illuminates DNA methylation variations in DS women, offering insights into potential protective factors against BC in DS.The prevalence of ER-negative (ER^-^) BC is higher in African American/Black women than in other US ethnic groups. A study by Chen et al. explored genome-wide DNA methylation in ER^-^ tumors, initially focusing on protein-coding genes and later delving into 96 differentially methylated loci (DMLs) in intergenic and noncoding RNA regions. Using Illumina Infinium Human Methylation 450K array and RNA-seq data, 23 DMLs were found to significantly correlate with the expression of 36 genes within a 1Mb radius. One hypermethylated DML (cg20401567) in ER^-^ tumors from Black women mapped to a potential enhancer downstream of HOXB2, indicating reduced HOXB2 expression. Independent analysis of 207 ER^-^ BC from TCGA confirmed this, suggesting that epigenetic disparities may influence BC pathogenesis in ER^-^ tumors between Black and White women.An interesting study explored olfactory receptors, specifically G protein-coupled surface receptors, that are increasingly relevant in carcinogenesis and metastasis. Their ectopic expression, influenced by environmental factors, can lead to methylation aberrations. This study identified 68 differentially methylated olfactory receptors in BC. Notably, hypomethylation events included BC signatures. Network analysis suggests a pivotal role of those receptors in stimulating metastasis-related pathways. Phenotypic smell tests revealed a generalized impairment in BC patients, independent of chemotherapy, highlighting olfaction’s crucial role in carcinogenesis. Olfaction receptors were shown as a potential factor of carcinogenesis in a well-characterized BC subset (Fessahaye et al.).

Recent advancements in genomics and other high-throughput biomolecular techniques, collectively referred to as “-omics,” have provided valuable insights into the molecular processes driving the development and progression of BC. Numerous mechanisms involved in these processes operate at multiple regulatory levels. The review article by Ochoa and Hernández-Lemus aimed to present a comprehensive overview of the current understanding of how various omics, such as DNA methylation, non-coding RNA, and other epigenomic changes, contribute to the regulation of BC. The molecular intricacies of multi-omic regulation in BC hold significant promise and could guide the development of innovative therapeutic strategies for BC. Additionally, a case report by Lv et al. presented a patient with primary ovarian and breast cancers, a condition with rising incidence due to the advances in early cancer detection. Using the technology of next-generation sequencing, a rare EGFR T790M mutation was detected in the patient’s primary BC tissue. A therapeutic recommendation with the targeted therapy “Osimertinib” was subsequently identified based on this mutation. In addition to the interesting case report, a mini-literature review was provided.

Several studies explored various angles of metabolic processes and their regulators in breast tumorigenesis. The case-control study by Zhou et al. investigated the impact of metabolic syndrome (MetS) on BC patients undergoing neoadjuvant chemotherapy (NAC). Among 221 female BC patients, 24.0% achieved pathologic complete response (pCR) after NAC. MetS was an independent predictor of lower pCR rates. Also, metabolic parameters, particularly blood lipid index, significantly worsened post-NAC. Over a 6-year follow-up, MetS was strongly linked to increased recurrence and mortality. The risk of death and disease progression rose with the number of MetS components. These findings suggest that MetS in BC patients undergoing NAC is associated with poorer outcomes. Another study focused on the challenges of triple-negative BC (TNBC) treatment, emphasizing the lack of therapeutic targets and poor prognosis. Using 16S rRNA MiSeq sequencing and metabolomic analysis on formalin-fixed, paraffin-embedded (FFPE) tissue samples, the research identifies Turicibacter’s higher abundance in TNBC, along with distinct metabolites. Significant correlations were found between intra-tumoral microbiome, clinicopathological characteristics, and HER2 expression. Microbial taxa associated with tumor-infiltrating lymphocytes suggest potential markers for antitumor immunity. The study’s innovative use of FFPE samples offers insights into diagnostic biomarkers, therapeutic strategies, and early TNBC clinical diagnosis (Wang et al.). Among various risk factors for BC, breast density and exposure to sex steroids are considered major ones. The research question posed by Ekstrand et al. was to explore whether those two key factors could affect extracellular space metabolism-regulating proteins, thus providing potential diagnostic and therapeutic markers. The investigators reported differentially expressed genes in both conditions and showed that two proteins, namely, pro-cathepsin H and galanin peptide, were similarly regulated in BC, dense- and estrogen-exposed breasts. The study underscores the potential role of metabolic proteins in better understanding the disease pathogenesis, diagnosis, and therapy. Furthermore, BC patients have been frequently observed with deranged lipid profiles and cholesterol metabolism. In the study conducted by Wu et al., an analysis of expression patterns of 73 cholesterol homeostasis-related genes was implemented on BC samples in the TCGA cohort with consensus clustering analysis. They used machine learning to compare multi-omics of different samples, aiming to predict the disease prognosis in different risk groups. The study could decipher the signature of cholesterol homeostasis-related genes for several key processes, namely, angiogenesis, immune responses, and therapeutic response.

## 2 Conclusion

In conclusion, the collective articles presented in this editorial, “*Epigenetic and metabolic regulators of breast carcinogenesis*,” underscore the intricate interplay between epigenetic mechanisms and metabolic dysregulation in breast cancer development. The findings from the contributed papers have illuminated various pathways and molecular mechanisms implicated in breast carcinogenesis. Moving forward, this comprehensive understanding offers promising avenues for future research endeavors, ranging from targeted therapeutic interventions to precision medicine approaches. This collection of articles not only enriches our current understanding of breast cancer pathogenesis but also serves as a catalyst for driving innovative research directions aimed at advancing diagnostics and prognostics and, ultimately, improving patient outcomes in this critical area of oncology.

## Author contributions

IT: Writing – original draft, Writing – review & editing. MS-A: Writing – original draft, Writing – review & editing. HB: Writing – original draft, Writing – review & editing. NE: Writing – original draft, Writing – review & editing.

